# Evolution of spatial and temporal patterns of railway container transportation: A case study of China cities

**DOI:** 10.3389/fpubh.2022.1087234

**Published:** 2022-12-14

**Authors:** Zhizhen Bai, Haibo Kuang, Jun Yang, Haijiang Li

**Affiliations:** ^1^School of Maritime Economics and Management, Dalian Maritime University, Dalian, China; ^2^Collaborative Innovation Center for Transport Studies, Dalian Maritime University, Dalian, China; ^3^Human Settlements Research Center, Liaoning Normal University, Dalian, China; ^4^Jangho Architecture College, Northeastern University, Shenyang, China

**Keywords:** railway container transportation (RCT), temporal and spatial pattern, evolution law, city scale, China

## Abstract

The healthy development of railway container transport is an important part of railway freight transport and is key for promoting the green transformation of China's transport and supporting a new pattern of transport development. In this study, railway container handling station (RCHS) data, kernel density analysis, standard deviation ellipse, Herfindahl–Hirschman index (HHI), trend surface analysis (TSA), and R index were applied to examine the evolution characteristics of container transport patterns after the market-oriented reform of China's railway freight transport in 2013. The results are as follows: First, the overall scale growth trend is obvious, and the double-center effect of transport scale on the Bohai Rim region and Chengdu–Chongqing Economic Zone is evident, with the transport center of gravity moving northward. Second, the amount of attraction/occurrence is consistent in spatial distribution, and the aggregation effect of both is similar, essentially exhibiting a tendency of being high in the northwest and low in the southeast. Third, the pattern of “export-oriented in the north and import-oriented in the south” has taken shape; nearly half of cities in China have stable traffic functions, stable traffic supply, and demand relationships, and the change of functions shows that the industrial structure is constantly upgrading. This study elucidates the pattern of railway container transport in cities in China and provides empirical guidance for adjusting the functions of urban freight transport, thereby promoting the healthy development of urban freight transport and optimizing urban transport planning.

## Introduction

Railway transport is an economical and environmentally friendly transport mode that is integral to any comprehensive transport system and an important logistics channel between cities (1, 2). It is also an important driving force for the healthy development of urban traffic. In 2021, the government of China included carbon peak and carbon neutrality in its policy reports for the first time. The transport sector of China was among the largest consumers of energy and worst carbon emitters (3). Therefore, adjusting the transport structure by increasing the railway transport proportion is essential for implementing the development concept and the “3060” carbon target (i.e., achieving carbon peak and carbon neutrality in 2030 and 2060, respectively) of China. In 2013, China Railway introduced various reforms, including supply-side structural reforms, freight rate adjustments, and railway company system reforms. However, by 2017, the total railway container transportation (RCT) volume was only 5.4% of the railway freight, far lower than the 30–40% level in developed countries (4). Developing RCT and adjusting transport are necessary not only to realize carbon peak and carbon neutrality but also to build a powerful transportation system in a country. Therefore, understanding how the evolution characteristics of RCT of China conform to the current development hot spots is conducive to adjusting the structure of urban freight transport and important to promoting the healthy development of urban transport and building an efficient logistics system.

In 2018, the National Development and Reform Commission and the Ministry of Transport jointly issued the “National Logistics Hub Layout and Construction Plan,” to promote a “shift from road to rail” and develop railway multimodal container transport to meet the modern demands of logistics (5). “Prospects and actions to jointly build the belt and road” emphasize the importance of building international land passage roadways (6), creating a new dimension in the development of China's RCT. In addition, owing to the geographical conditions and unbalanced distribution of social and economic factors, RCT will become the primary long-distance transport mode and the main carrier of the Silk Road Economic Belt. As the principal link of multimodal transport, RCT will become a more competitive choice for medium- and long-distance freight transport. Analyzing the temporal and spatial evolution characteristics of RCT is of great significance to find urban traffic hubs and to promote the rational allocation of traffic resources and the healthy development of urban traffic.

The purpose of this study is to elucidate the differentiation pattern and agglomeration effect of RCT in China, as well as the functions and changes of urban freight transport. This article discusses the change in the container transport scale and center of gravity of China's railway based on the visualization of container loading and unloading station data, by utilizing kernel density analysis and standard deviation ellipse. The HHI and trend surface method were used to calculate the distribution effects at different scales. To build on the results, a location quotient was used to analyze the division and transformation of freight functions. The novelty of this study is that it adopts more accurate data of railway container loading and unloading stations, which differ from traditional statistical data, and elucidates the temporal and spatial evolution modes of RCT traffic on the city scale.

The remainder of this article is organized as follows: Section Literature review summarizes relevant research on the RCT. Section Research data and methods presents the data and methods used. Section Results and analysis describes the differentiation law of RCT in China. Section Discussion discusses the findings. Section Conclusion concludes this study.

## Literature review

Improving transportation efficiency and promoting its high-quality development have always been key considerations in RCT.

Close attention should be given to the mode of transportation. Freight and environmental costs can be decreased by increasing the market share of RCT and promoting the shift from road to rail. This entails optimizing container station locations (7), improving the operational efficiency (8), and enhancing the service quality (9), which can help achieve a balance between RCT resources and benefits. Researchers have promoted the development of sea–rail combined transport (10) by examining the relationship between railway containers and terminals (11) to accelerate the shift from road to rail. Some studies have focused on upgrading and optimizing RCT equipment, including the container safety equipment chain (12), gantry crane dispatcher (13), and tank containers (14) to improve transport efficiency. The low environmental cost of RCT is primarily reflected in its energy consumption and CO_2_ emissions. Increasing the road–rail combined transport ratio (15) and optimizing the location of the central station (16) are effective measures to preserve energy and reduce emissions. The mode of transportation includes transportation equipment, transportation station location, and transportation efficiency as the primary visual factors to realize the high-quality development of RCT; however, improving the transportation quality according to the transportation activities rules is typically neglected.

The spatial relationship with social economy determines the differences in research emphasis. India has expanded the volume of RCT by altering its RCT policies (17) to foster and favor sustainable development of freight transport (18). The rail freight reform of Turkey has increased the freight demand (19) and focused on the relationship between freight growth and economic growth (20). The completion of China's “eight vertical and eight horizontal” railway network (21) and the speeding up of railways will promote the polarization of the railway freight space (22). The growing container freight market will also have a significant impact on the inter-regional freight transportation pattern, profitable division of the labor pattern, and industrial layout (23). The radiation of the effect of hub sites on the hinterlands (24–26), operation plan optimization (27), and freight capacity improvement (28) are key research problems in RCT. Furthermore, the “high-speed rail effect” (29–31), China Railway Express platform-seeking cooperation (32), greenhouse gas emission reduction (16), China–Europe container transport (33), and railway container multimodal transport (34) have become the main topics of freight transport research. In the field of railway and urban research, railway traffic flow has been a popular topic in the hybrid research on traffic geography and urban geography. From the 1990s, Jin Fengjun, et al. have been focusing on the inter-provincial railway freight transport pattern (35, 36). Later, with the development of railway transport, the focus has expanded from the railway network connection (37) to the container exchange hub and its logistical features (38). Meanwhile, the longitudinal organization structure of container flow was refined to the city level (39), and research coverage was gradually expanded. Special commodities such as coal (40, 41) and special regions such as Northeast China (22, 42) and the Yangtze River Delta (43) have also become important parts of railway transport research. Previous studies have examined the steps necessary to promote economic development through RCT activities, focusing on the impact of transportation activities on the economy and emission reduction; however, there are still some problems in understanding the transportation rules. Although big data have been widely used in the field of urban transportation (44, 45), statistical data continue to be the most commonly used data in railway container transport research. Compared with statistical data, the railway container loading and unloading station data used in this study have finer granularity and higher accuracy.

Previous research covers a wide range of problems, including optimizing the transportation mode, macrohub stations, operation schemes, multimodal transport, and other problems that depend on regional factors. Furthermore, several studies have focused on social and economic development; their research methods are mainly based on mathematical modeling, and the research data include statistical data and passenger and freight websites. Moreover, the research scale is mostly restricted to the regional and provincial levels. However, there are still some issues, such as insufficient research on the law of RCT, rough research scale, and inaccurate research data. Understanding the transportation scale and movement of the center of gravity of transportation activities, exploring its evolution law and agglomeration effect, and analyzing the transformation of city-scale transportation functions are conducive to comprehending the law of RCT activities from macro- and micro-perspectives, providing a realistic basis for modifying the city freight transportation functions, and optimizing the allocation of railway container resources. Therefore, the development of RCT conforms to China's transportation structure adjustment plan involving “changing bulk transportation into container transportation, turning to the railway” and the goal of “peak CO_2_ emissions and carbon neutrality.” This study uses the data of railway container-handling stations to understand the law of RCT activities on a city scale and provide suggestions for the healthy development of urban transportation.

## Research data and methods

### Research data

**Figure 1:**
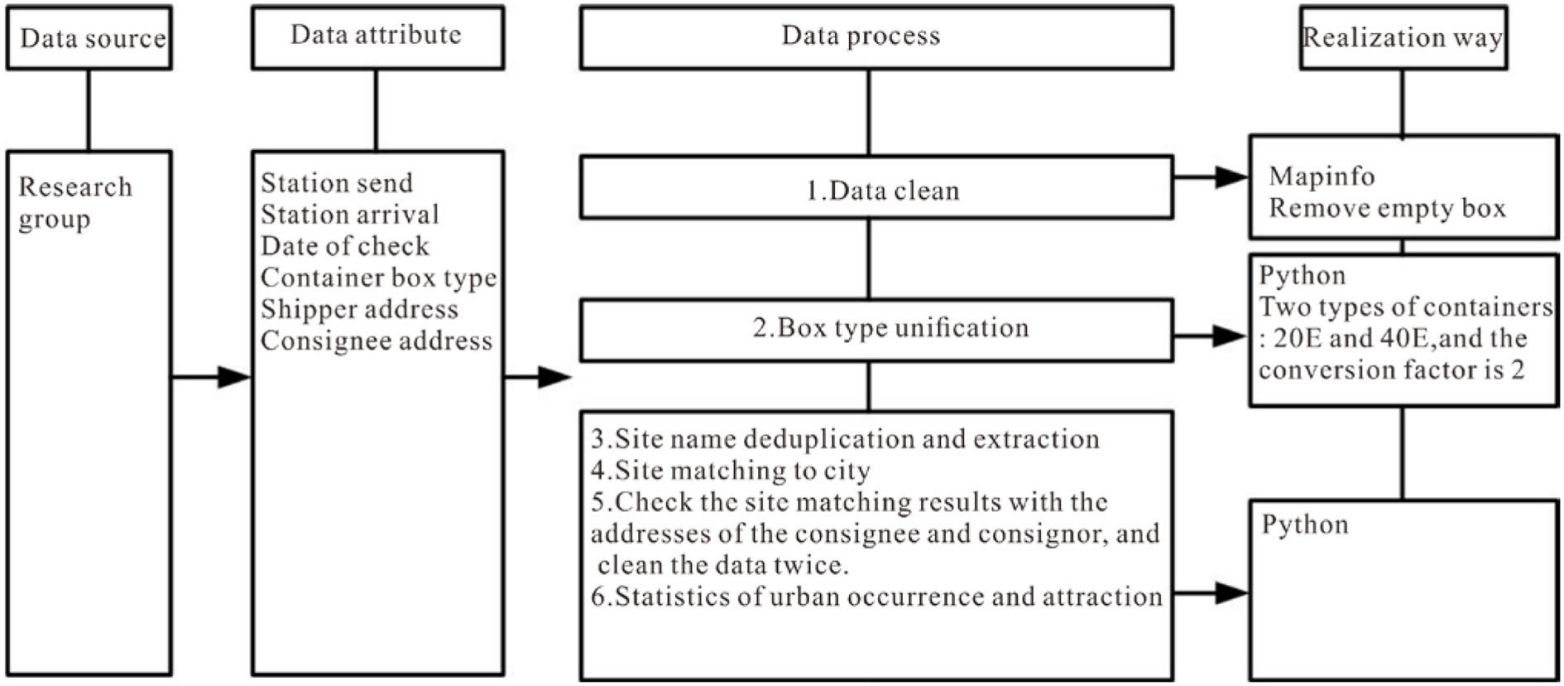
Data and basic processing.

This study explores the law of railway container transport in China after the railway freight reform using RCHS data from 2013 to 2020. These data include the inland transport sections of China–Europe trains, but not include the date of departure and destination stations outside China. The granularity of the study is national prefecture-level administrative units, and the basic attributes of the data are shown in Table 1. The data are cleaned, extracted, and coefficient-converted, and duplicated data are deleted (Figure 1). Hong Kong, Macau, and Taiwan provinces are not included in this study.

**Table 1:** Basic attributes of research data.

**Year**	**Number of stations**	**Number of cities**	**Year**	**Number of stations**	**Number of cities**
2013	563	249	2017	1,076	288
2014	593	251	2018	1,207	302
2015	662	267	2019	1,326	306
2016	872	275	2020	1,529	314

**Figure 2:**
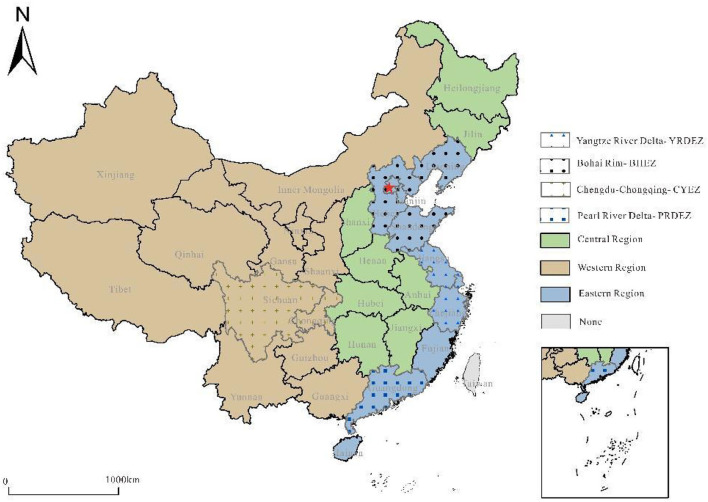
Research area.

Geographical features and resource endowments cause significant variations in China's economic structure and level of transport development. The differences in location and natural conditions in western, central, and eastern regions result in significant differences in economic development and traffic levels. By considering a large area as a unit, the agglomeration level of China's railway freight can be examined from a macro-perspective. The freight level of the most developed area in China can be described from the perspective of important economic zones (Figure 2).

### Research method

#### The scale of traffic and the shift of center

(1) Kernel density analysis (KDA)

The kernel do describe the scale characteristics of RCT and to determine the dense areas of RCT in China. Considering the city as the base point and the traffic volume as the weight, the traffic scale characteristics are obtained, as follows:


(1) $$\begin{array}{l} f\left(x\right)=\frac{1}{nh}\overset{n}{\underset{i=1}{\sum }}k\left(\frac{x-x_{i}}{h}\right), \end{array}$$

where *n* is the number of cities; *k* is the weight function; *h* is the bandwidth, that is, the surface width extending in space with *x* as the origin; and *x–x*_*i*_ is the distance between the density estimation points *x* and *x*_*i*_.

(2) Elliptical standard deviation (ESD)

This method is used to analyze the main direction and distribution range of the RCT scale, including the center of gravity, long and short semi-axes, orientation, and other factors. Considering the transport volume as the weight, the ESD center of gravity is used to describe the change in the center of gravity of RCT in the study period:


(2) $$\begin{array}{l} \bar{l}=\frac{\sum_{i=1}^{n}l_{i}}{n},\bar{L}=\frac{\sum_{i=1}^{n}L_{i}}{n}, \end{array}$$

(3) $$\begin{array}{l} \tan=\\ \frac{\left(\sum_{i=1}^{n}{\tilde{l}}_{i}^{2}-\sum_{i=1}^{n}{\tilde{L}}_{i}^{2}\right)+\sqrt{{\left(\sum_{i=1}^{n}{\tilde{l}}_{i}^{2}-\sum_{i=1}^{n}{\tilde{L}}_{i}^{2}\right)}^{2}+4{\left(\sum_{i=1}^{n}{\tilde{l}}_{i}{\tilde{L}}_{i}\right)}^{2}}}{2\sum_{i=1}^{n}{\tilde{l}}_{i}{\bar{L}}_{i}}, \end{array}$$

(4) $$\begin{array}{l} \sigma_{l}=\sqrt{2}\sqrt{\frac{{\sum_{i=1}^{n}\left({\tilde{l}}_{i}\cos\theta -y_{i}\sin\theta \right)}^{2}}{n},} \end{array}$$

(5) $$\begin{array}{l} \sigma_{L}=\sqrt{2}\sqrt{\frac{{\sum_{i=1}^{n}\left({\tilde{l}}_{i}\sin\theta +L_{i}\cos\theta \right)}^{2}}{n},} \end{array}$$

where li and Li are the spatial location coordinates of each city; li and Li are the arithmetic average centers; n is the number of cities; θ is the elliptical azimuth; σl and σL are the standard deviations along the X and Y axes, respectively; and ${\tilde{l}}_{i}$ and ${\tilde{L}}_{i}$ are the distances from the center of gravity of the coordinates.

#### Evolution of agglomeration effect

(1) Herfindahl–hirschman index (HHI)

Agglomeration reflects the spatial imbalance between resource elements and economic activities (46). The HHI is used to measure the degree of agglomeration of spatial elements (47, 48). The RCT concentration level is discriminated by the HHI, expressed as follows:


(6) $$\begin{array}{l} {HHI}_{i}=H{HI}_{i}=\overset{n}{\underset{i=1}{\sum }}{\left(\frac{x_{i}}{T}\right)}^{2}, \end{array}$$

where *x*_*i*_ is the import/export volume of RCT in city *i, T* is the total export/import volume of RCT in the province (region or typical economic zone) where the city is located, and the *HHI*_*i*_ lies in the range [0,1]. The closer it is to 1, the higher is the concentration of RCT. If there is only one city at the municipal and provincial levels, the *HHI*_*i*_ is 1.

(2) Trend surface analysis (TSA)

In the equation for the simulation of the spatial differentiation of the agglomeration effect by TSA, *Z*_*i*_ *(l*_*i*_*, L*_*i*_*)* is the *HHI* of city *i*, (*l*_*i*_*, L*_*i*_) is the geographic coordinates of the city *i*, and the *X* and *Y* axes represent the east–west and north–south, respectively.


(7) $$\begin{array}{l} Z_{i}\left(l_{i},L_{i}\right)=F_{i}\left(l_{i,}L_{i}\right)+\varepsilon_{i}, \end{array}$$

where *F*_*i*_*(l*_*i*_*,L*_*i*_*)* is the fitting value of the trend surface and ε_*i*_ is the related random disturbance term indicating the error between the real value and the trend value of the RCT in city *i*. The trend value is calculated by a second-order polynomial, as follows:


(8) $$\begin{array}{l} F_{i}\left(l_{i},L_{i}\right)=\beta_{0}+\beta_{1}l+\beta_{2}L+\beta_{3}l^{2}+\beta_{4}{yL}^{2}+\beta_{5}lL, \end{array}$$

where *l*^2^ and *L*^2^ indicate that the highest degree of geographical coordinates is 2, and β_0_*-*β_5_ are the coefficients of the polynomial.

#### Evolution of transport function

(1) Location quotient (LQ)

The LQ was used to describe the advantage of each city in the RCT system. It is calculated as the ratio of the container output/input ratio of a city to that of the whole country:


(9) $$\begin{array}{l} Q_{ie}=\frac{\frac{x_{ie}}{\left(x_{ie}+x_{if}\right)}}{\frac{\sum_{j=1}^{m}T_{je}}{\sum_{j=1}^{m}\left(T_{je}+T_{if}\right)}}, \end{array}$$

(10) $$\begin{array}{l} Q_{if}=\frac{\frac{x_{if}}{\left(x_{ie}+x_{if}\right)}}{\frac{\sum_{j=1}^{m}T_{je}}{\sum_{j=1}^{m}\left(T_{je}+T_{if}\right)}}, \end{array}$$

Where *Q*_*ie*_ and *Q*_*if*_ Represent the Export Location Entropy and Import Location Entropy, Respectively; *x*_*ie*_represents the export volume of RCT in the city *i*; *x*_*if*_ represents the import volume of RCT in the city *i*; *T*_*je*_ represents the province container export volume; *T*_*jf*_ represents the province container import volume; *n* is the number of cities; and *m* is the number of provinces.

(2) R_i_ index

RCT is an economic activity with vector characteristics. The relationship between freight import and export reflects the relationship between supply and demand in urban RCT. The *R*_*i*_ index was developed to determine the basic functions and changes in RCT in a city, as follows:


(11) $$\begin{array}{l} R_{i}=\frac{x_{ie}}{\left(x_{ie}+x_{if}\right)}, \end{array}$$

where *x*_*ie*_ is the export volume of RCT in the city *i, xif* represents the import volume of RCT in the city i, and the range of *R*_*i*_ is [0,1]. The closer the value is to 1, the higher is the RCT export level.

## Results and analysis

### Overall scale and center of gravity shift of RCT in China

#### Evolution of total scale of RCT

**Figure 3:**
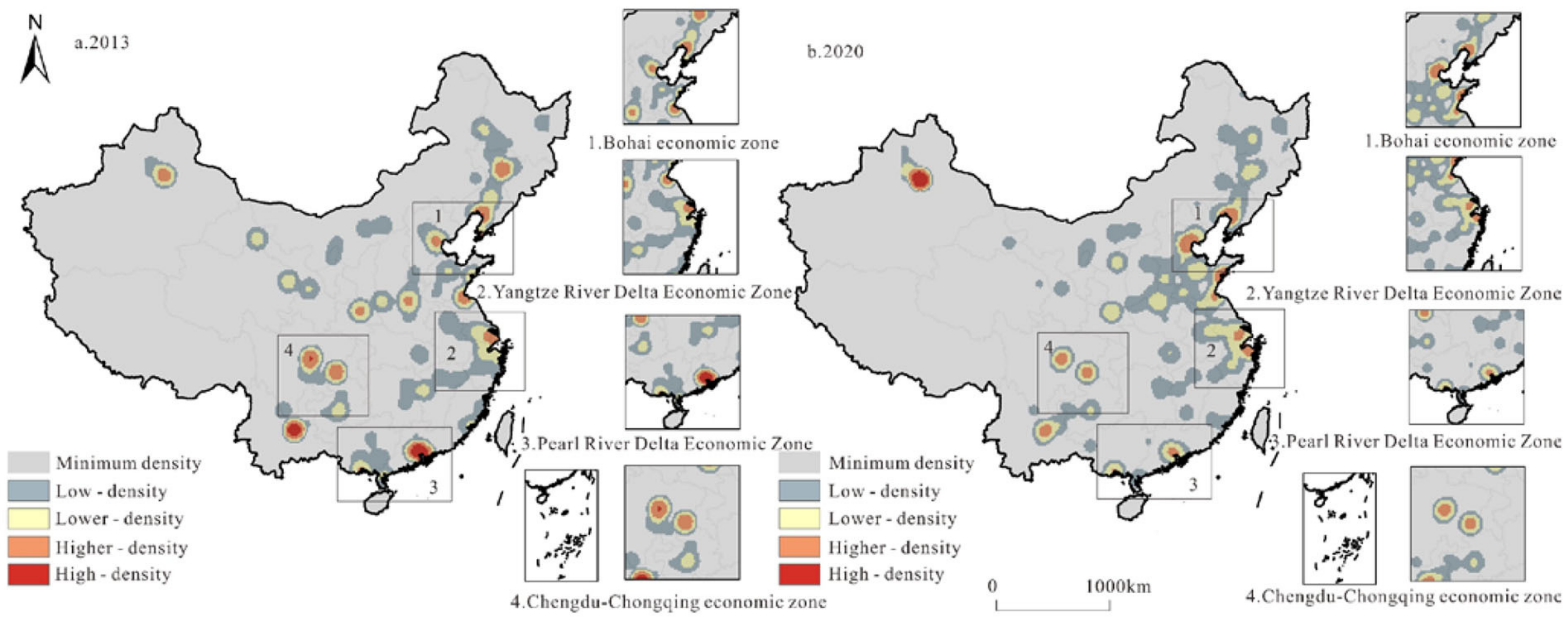
Evolution of the total scale of railway container transport in China.

The first year and last year of the study period are chosen as representative years to describe the overall scale of the evolution of RCT (Figure 3). Overall, the transport scale in 2020 is much higher than that in 2013, and the flaky characteristics gradually appear, especially in the Bohai Rim region. Urumchi, Kunming, Zhengzhou, Xi′an, and other cities are high-value areas in western, southwestern, and central regions, and they are all container transport hub cities. From the perspective of four typical economic zones (Bohai Rim, BHEZ; Yangtze River Delta, YRDEZ; Pearl River Delta, PRDEZ; Chengdu–Chongqing, CYEZ), the dual-center effect of the transport scale in Bohai Rim is obvious (Tianjin–Yingkou and Dalian). Meanwhile, the Yangtze River Delta Economic Zone has the estuary as the center, showing circular diffusion. The opening of China–Europe trains attracted the original supply of goods, which led to a sharp drop in the transport scale in the Pearl River Delta, and the Chengdu–Chongqing economic zone has the highest transport scale. The moving range of the transportation center of gravity is small, and the trend of moving to the north is obvious. The direction of evolution is northwest, northeast, southwest, northwest, and southeast, but the scope is basically concentrated in Henan Province, which is closely related to the intersection of the Longhai Line and Beijing–Guangzhou line here. The Longhai–Lanxin Line and Beijing–Guangzhou Line are important east–west and north–south railway lines in China, and the change in transport centers in this range shows that the railway network is the decisive factor of freight transport.

#### Shift of center of gravity of container transport

**Figure 4:**
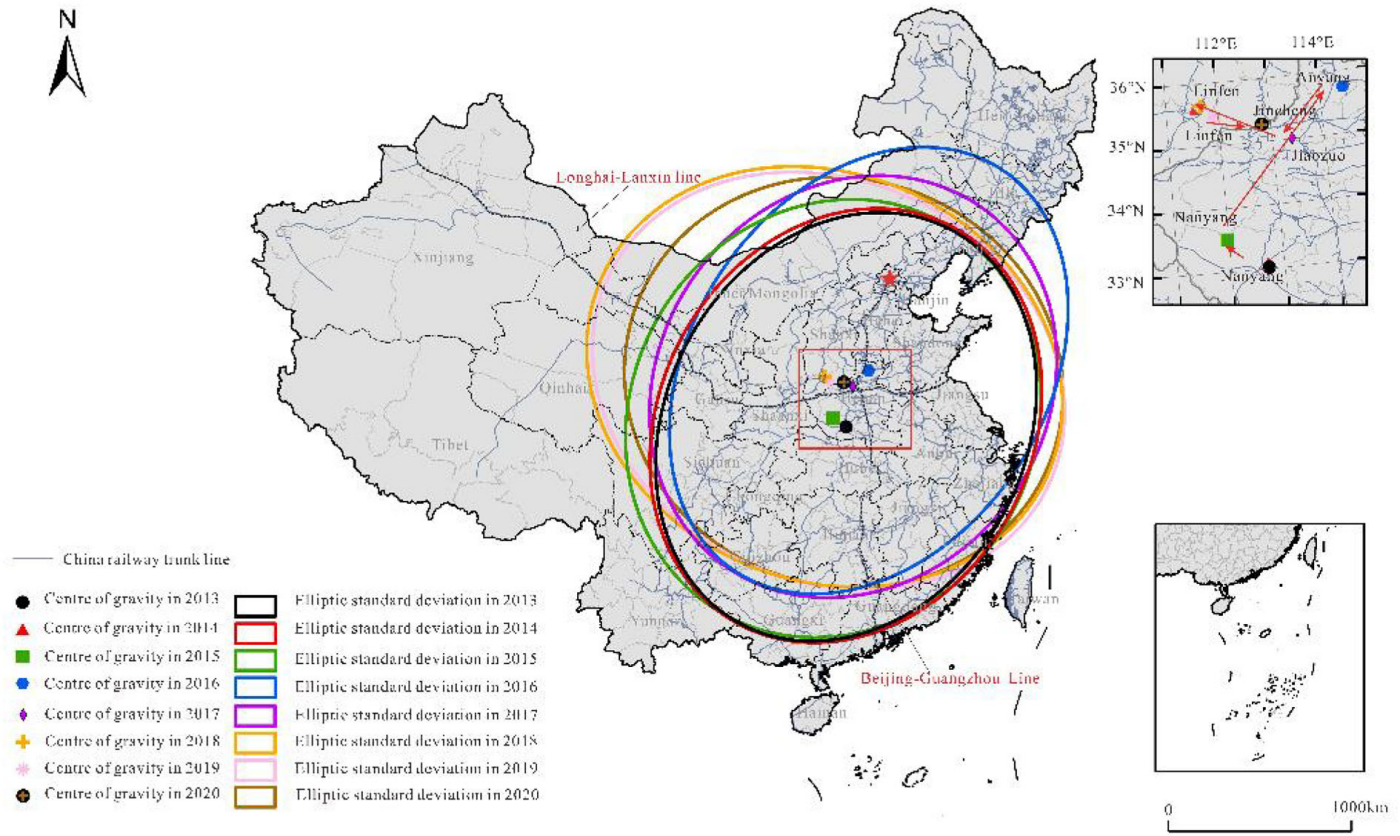
Shift of center of gravity of China's railway container transport.

During the research period, the movement of the center of gravity of railway container transport is characterized by the standard deviation ellipse (Figure 4). Influenced by the opening of China–Europe trains, the distribution direction of transport has changed from northeast to southwest to northwest to southeast, and the northwest has become the main area of RCT. The movement of the center of gravity has not changed even slightly, and the trend of moving to the north is obvious. The direction of evolution is northwest, northeast, southwest, northwest, and southeast, but the scope is basically concentrated in Henan Province, which is closely related to the intersection of the Longhai Line and Beijing–Guangzhou line here. The Longhai–Lanxin Line and Beijing–Guangzhou Line are important east–west and north–south railway lines in China, and the change in the transport center in this range shows that the railway network is the decisive factor of freight transport.

### Evolution and agglomeration effect of China's RCT

#### Evolution of RCT export and import patterns

**Figure 5:**
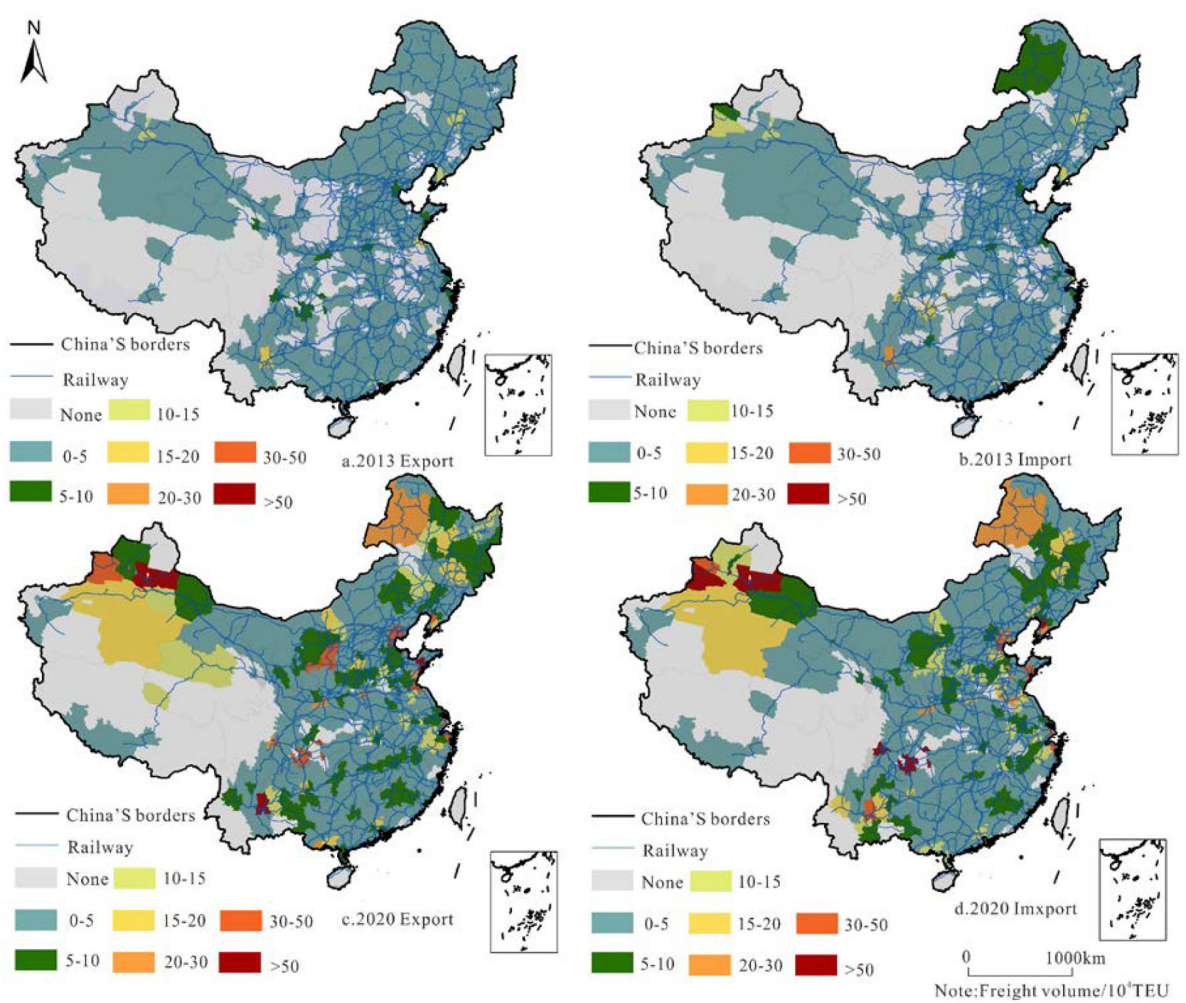
Export/import pattern and evolution of China's railway container freight in 02013/2020.

A railway container is a vector transport mode of scattered goods between cities, and its occurrence/attraction mode reflects the development level of urban RCT and the relationship between supply and demand (Figure 5).

The occurring/attracting volume relates to the outward and inward cargo transport through railway container transport in the following ways. 1. Container transport has an evident growth trend, and the occurrence/attraction amount is consistent in spatial distribution. Tianjin, Changchun, Dalian, Shanghai, Chongqing, Kunming, and other cities are located at the forefront of the country in terms of incidence and attractiveness; 2. the container transport volume of major railway trunk lines and cities near hubs is relatively high, such as in Changchun where Beijing—Harbin transits, Tianjin where Beijing–Harbin meets the Beijing–Shanghai railway, and Kunming where the Cheng–Kun, Guizhou–Kunming, and Nanning–Kunming lines meet. These are typical high-value areas of railway container transport in China; and 3. the number of high-volume container transport cities has increased substantially, and in recent years, China's railway container transport development has gained good momentum. The number of cities with a high incidence (>100,001 TEU) increased from 8 to 50 and that with high attraction (>100,001 TEU) increased from 11 to 55. The number of low-income cities and low-attraction cities has declined significantly.

#### Spatial agglomeration of RCT at different scales

(1) Distribution trend of HHI of RCT volume

The TSA was fitted to analyze the agglomeration law of RCT at the provincial scale based on the 2013/2015/2017/2019 node.

**Figure 6:**
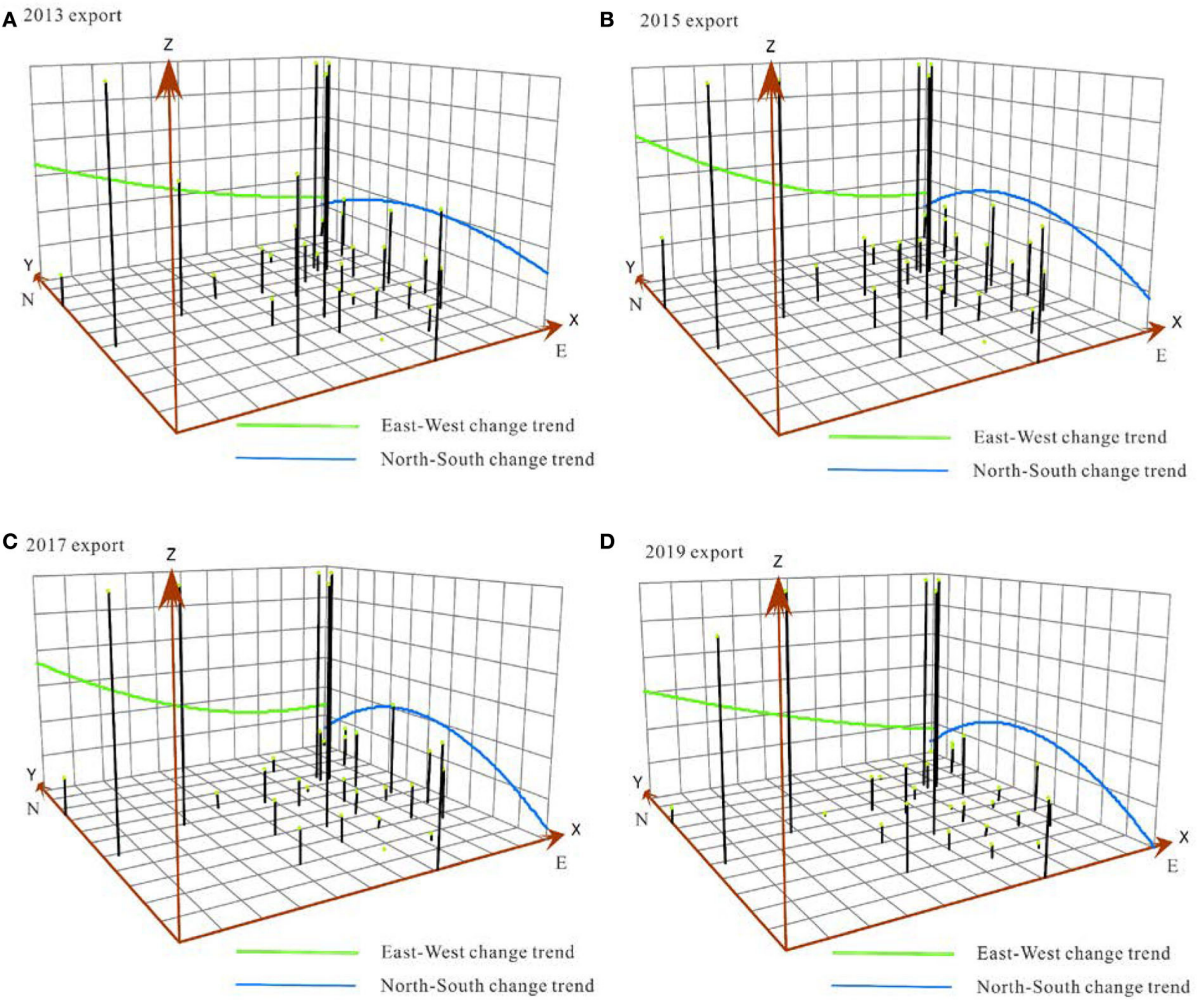
Spatial change trend of the HHI of railway container freight at the provincial scale (export).

**Figure 7:**
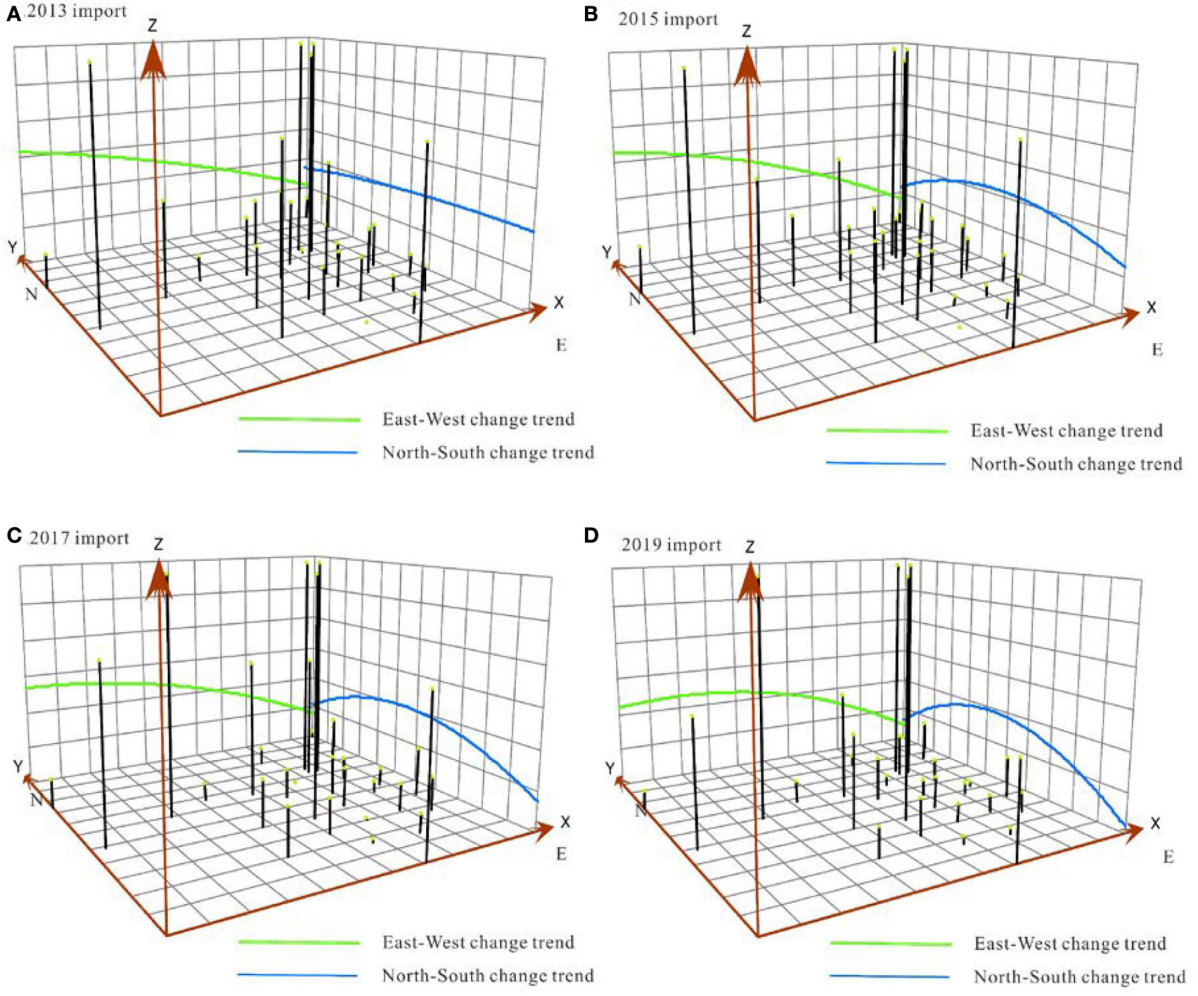
Spatial change trend of the HHI of railway container freight at the provincial scale (import).

The trend of the export agglomeration effect is as follows: The agglomeration effect in the north–south direction is arched, the difference in time series is increasingly obvious, and the agglomeration effect in East China is the highest; the degree of aggregation in the east–west direction gradually decreases, and the difference in time sequence is not significant. The agglomeration effect of railway container export in China basically exhibits a trend of being higher in the northwest and lower in the southeast (Figure 6). The trend of the import agglomeration effect is almost identical to that of the export agglomeration effect (Figure 7). In the north–south direction, the arch feature becomes more apparent, and the import agglomeration effect in East China is enhanced; in the east–west direction, the agglomeration effect gradually weakens from the west to east, and an arc-shaped trend gradually emerges. The tendency of China's railway container export agglomeration effect is similar to that of the import agglomeration effect.

(2) Agglomeration effect in eastern, central, and western regions

**Figure 8:**
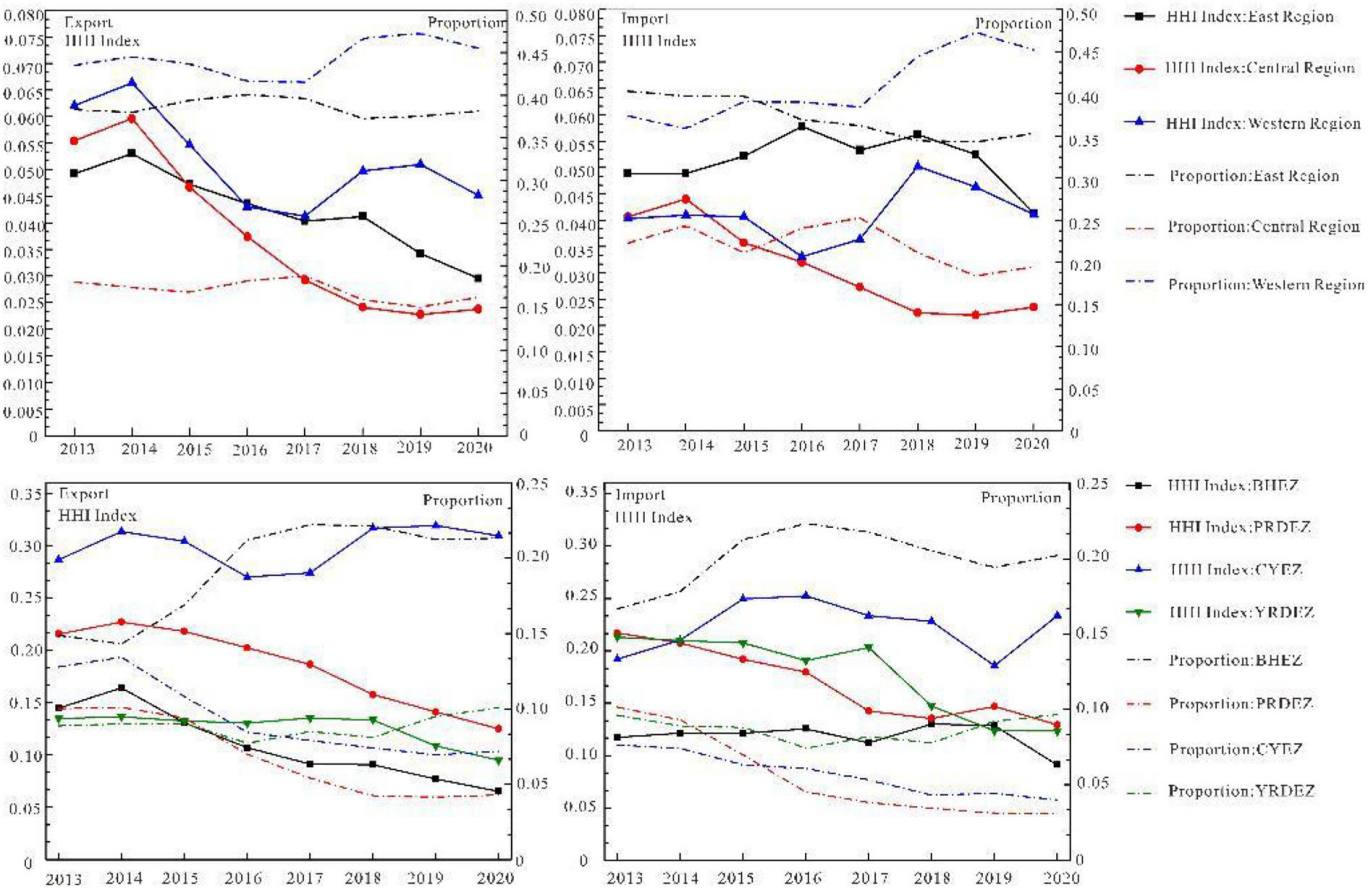
Evolution of agglomeration effect in eastern, central, western regions and important economic regions.

According to the division of the three major economic zones in the Seventh Five-Year Plan (49) and the important economic zones in China (BHEZ, YRDEZ, PRDEZ, and CYEZ), this study analyzes the agglomeration effect of the large areas and important economic zones (Figure 8).

The concentration status of RCT import and export strongly varies at the regional scale in the following ways: 1. the attractive concentration of RCT import is declining, and regional development is becoming more and more balanced. The western region holds the highest proportion of attracted traffic, floating around 40%, with the highest concentration, falling from 0.066 to 0.045. The central region accounts for the lowest proportion, about 17%. The concentration level of RCT has dropped sharply to 0.023 since 2014. The proportion in the east is about 39%, and the concentration ranges from 0.049 to 0.029. 2. The aggregation degree of RCT export exhibits a decreasing trend. The proportion is about 40 % in the west, and the concentration varies from 0.040 to 0.041, accounting for about 22% in the middle part; furthermore, the degree of agglomeration has decreased from 0.041 to 0.203. The proportion of the eastern region has dropped from 40 to 35%, and the concentration dropped from 0.048 to 0.041.

BHEZ, YRDEZ, PRDEZ, and CYEZ (50, 51) are important economic zones. RCT has a large market demand, with a huge demand in the transport market, with its occurrence and attraction accounting for 39 and 44%, respectively, of the country′s total. The attraction and incidence of BHEZ rank first, but its agglomeration level is at the bottom, and its container transport is the most balanced. CYEZ has the highest degree of attraction and occurrence, which is less than that of the Bohai Rim and Yangtze River Delta. The proportion and concentration of PRDEZ transport volume are less than those of the other three regions; therefore, its RCT should be strengthened. The occurrence proportion and agglomeration level of the YRDEZ are higher than the attraction, which indicates that container exports are the main export of the YRDEZ.

### Functional differentiation of China's RCT

#### Functional differentiation of RCT

**Figure 9:**
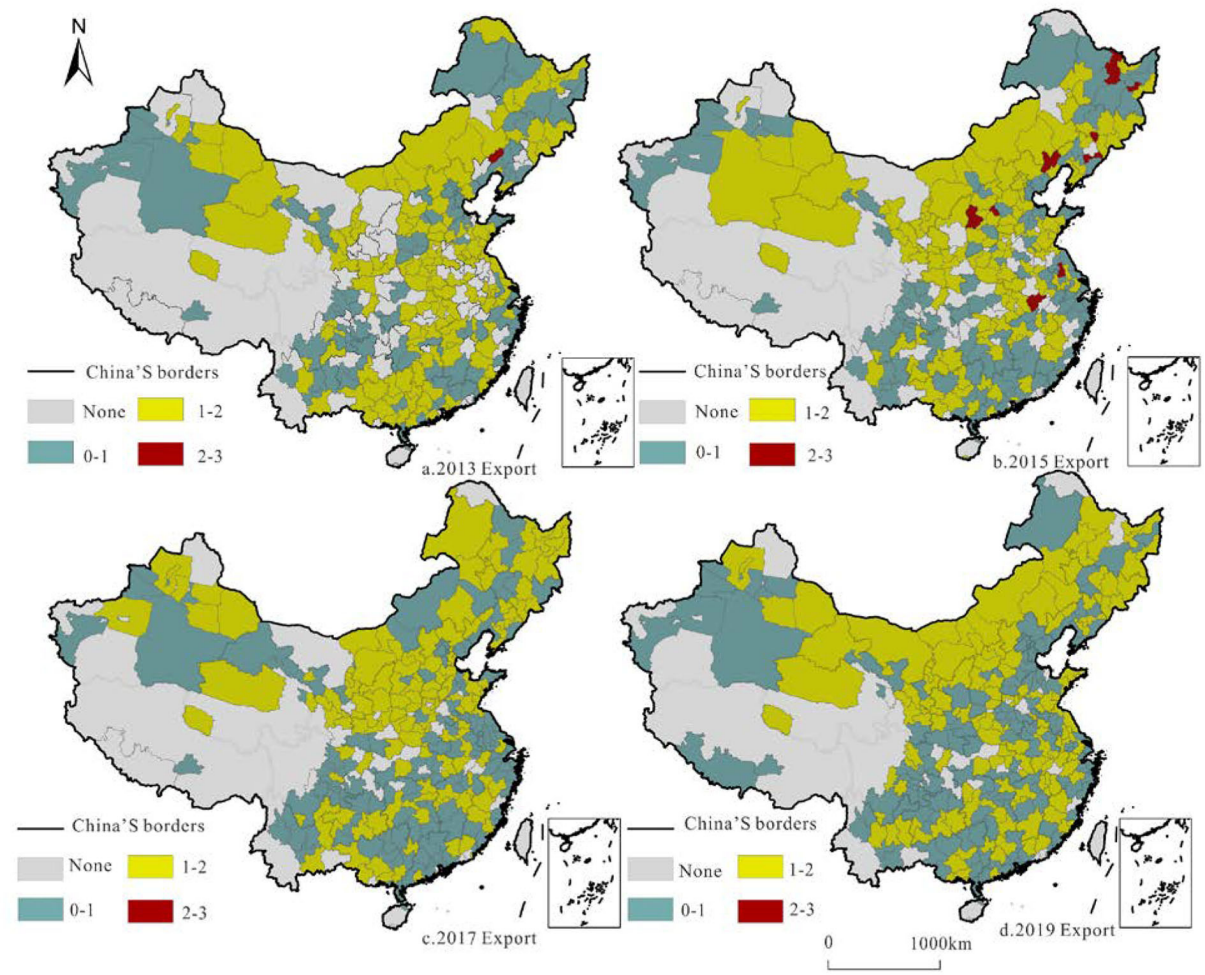
Location quotient pattern and evolution of China's railway container export volume in 2013/2015/2017/2019.

**Figure 10:**
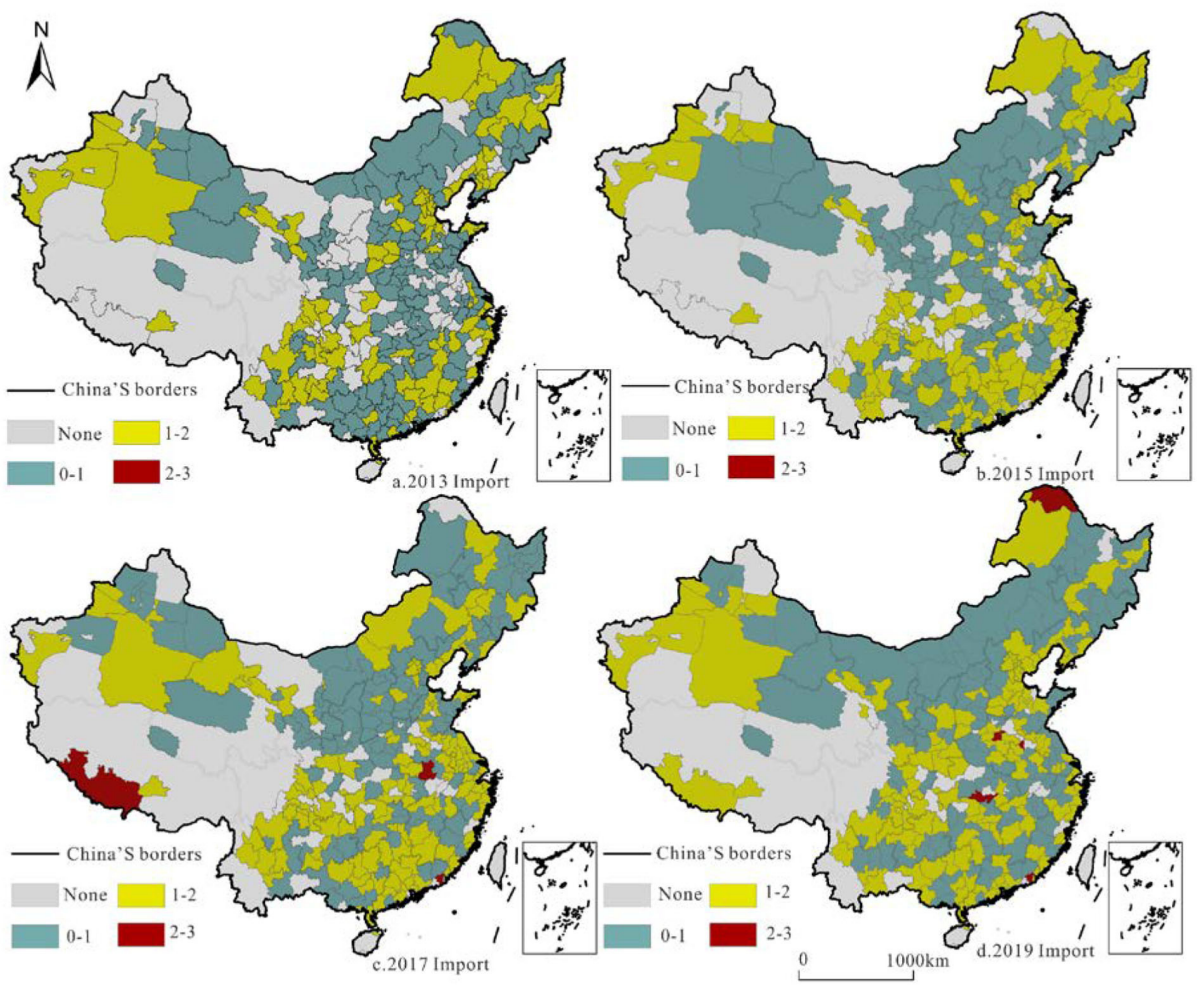
Location quotient pattern and evolution of China's railway container export volume in 2013/2015/2017/2019.

RCT strength reflects the container transport specialization and significantly impacts the freight transport base and supply capacity (Figures 9, 10). We calculated the LQ of railway container freight in each city and selected the representative years of 2013/2015/2017/2019 to determine its leading position in China.

The container export LQ reflects the dominant position of cities with RCT and carries the foreign freight supply capacity. Macroscopically, because the north has more resources than the south, and the industrial structure is characterized by the processing industry, the north and the south have distinct export LQs, especially in the central part of Inner Mongolia. The export LQs of Fuxin, Luliang, Yangquan, Benxi, Yichun, and other cities are more than 2, which is higher than the average, clearly demonstrating the advantage of freight export; however, these advantages have decreased.

The LQ of container import reflects the advantageous role of each city in undertaking internal and secondary regional freight forwarding in railway freight transport. In contrast to the distribution of the export LQ, the import LQ of southern cities is higher than that of northern cities, and it is becoming increasingly obvious. Daxing′anling, Shigatse, and other places where the import LQ is >2 are located in the border areas, with container transport as the main input.

#### Evolution of basic functions of RCT

**Figure 11:**
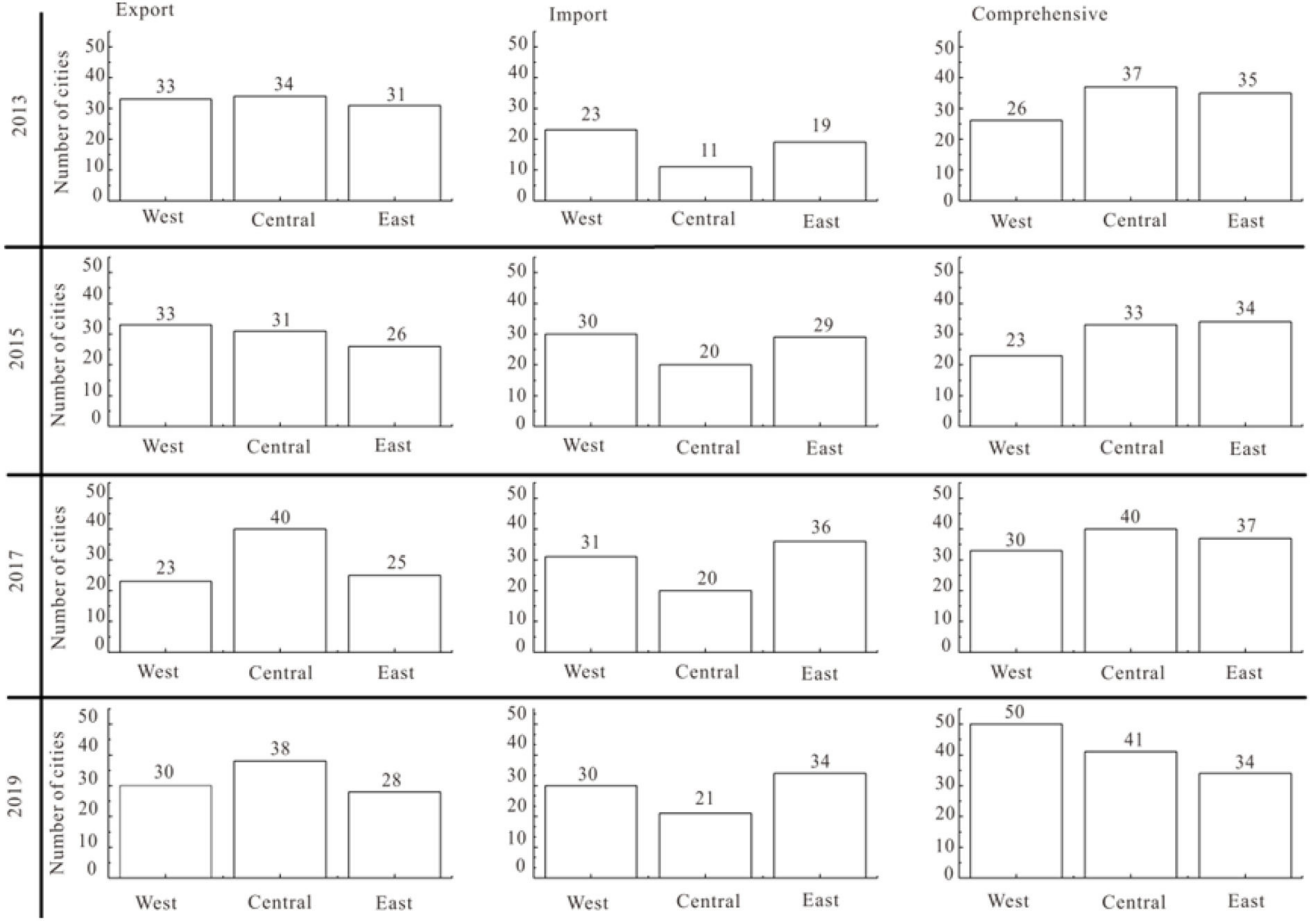
Types of railway container freight in different cities from 2013 to 2020.

The relationship between the RCT import and export determines its basic functions in freight transport systems. The *R*_*ij*_ index was constructed, and the elementary functions of the city were divided into input types (< 0.4): comprehensive (0.4–0.6) and output (>0.6). We used 2013/2015/2017/2019 as the representative years for surveying and mapping (Figure 11).

During the research period, the increase in sample cities meant that the number of the three types of cities increased, with comprehensive cities increasing the most. At the regional level, the different types of cities are ranked by quantity as follows: comprehensive >export >import. RCT is balanced in most cities in China. From the change in the number of various cities, the number of export-oriented cities in the western region decreased, while the number of import-oriented and comprehensive cities increased, which is consistent with the situation that scattered goods in the western region basically depended on imports. The number of types of cities in the central region was essentially stable, which is related to the industrial structure of the central region, indicating that it had to import resources from the western region and eastern region for processing and transport. In the eastern region, the export-type decreased, the import-type increased, and the comprehensive-type was basically stable. The eastern region was dominated by foreign trade, and it needed to import manufactured goods from the central and western regions, so the type of import increased.

In Table 2, *n* and *N* are the start and end years of the data, respectively. Among the changes in urban functions, 46% of the cities have a common traffic function, and nearly half of cities in China have stable traffic functions and stable traffic supply–demand relationships. The proportion of cities that have changed from export type to import type or comprehensive type is 24%, and the proportion of cities that have changed from import type to export type or comprehensive type is 12%. The industrial structure of these cities has changed, with the former being upgraded from the secondary industry to the tertiary industry, while the latter has improved from the original primary industry to the secondary industry. The change in the municipal RCT function shows that China's urban transport function is essentially stable, and the industrial structure is continuously upgrading.

**Table 2:** Evolution of the functions of China's railway container freight from 2013 to 2020.

**City type**	**West**	**Central**	**East**	**Total**	**%**	**Definition**
Always export	21	23	12	56	18	More than two-thirds of the *R_*ij*_* is >0.6
Always import	15	11	11	37	12	More than two thirds of the *R_*ij*_* is below 0.4
Always comprehensive	10	22	19	51	16	More than two thirds of the *R_*ij*_* is between 0.4 and 0.6
From export to import	15	11	12	38	12	*R_*ijn*_* >0.6, *R_*ijN*_*<0.6
From export to comprehensive	16	12	11	39	12	*R_*ijn*_* >0.6, 0.4<*R_*ijN*_*<0.6
From import to comprehensive	8	3	7	18	6	*R_*ijn*_*<0.4,0.4<*R_*ijN*_*<0.6
From import to export	5	7	7	19	6	*R_*ijn*_*<0.4, *R_*ijN*_* >0.6.
From comprehensive to export	8	12	6	26	8	0.6<*R_*ijn*_*<0.6, *R_*ijN*_* >0.6.
From comprehensive to import	15	4	13	32	10	0.6<*R_*ijn*_*<0.6, *R_*ijN*_*<0.4
Total	113	105	98	316	1	

## Discussion

### Impact of RCT on China's freight transport

With the gradual diversion of high-speed rail to railway passenger transport, the freight capacity of the railway is also constantly improving. Railway freight transport in China also exploits the potential of railway freight transport to improve the market share of RCT. Furthermore, RCT is of great significance to promoting the green transformation of China's transport, reducing the social logistics cost, and supporting a new pattern of transport development. Therefore, expanding railway freight transport and developing container transport are not only in line with the needs of social development but also in line with the policy orientation of the state (4). At present, the scale of China's RCT is gradually expanding, with its development gaining sound momentum. Influenced by the opening of China–Europe trains and the construction of the Great Passage in Northeast Asia, the transport center is gradually moving to the north. The distribution of resources, industrial division of labor, and structure play a decisive role in the evolution of the RCT agglomeration effect. It was observed that the traffic function of most cities in China is relatively stable, and the supply and demand of scattered commodities in these cities are essentially stable. The traffic functions of some cities have also changed, and the situation of RCT can reflect the change in the regional industrial structure to a certain extent.

Existing RCT studies pay more attention to the network structure (23), flow effect (33), and hub effect (52) and use statistical data to investigate the interaction among regions. There is a dearth of research on the extent of RCT scale, RCT agglomeration, and urban freight transport function. Compared with traditional statistical data, the data of railway container-handling stations can not only truly reflect the current situation of RCT in China but also help in more accurately analyzing its development, exploring its driving mechanism, and providing a scientific basis for optimizing container resource allocation.

### Limitation

This article describes the temporal and spatial evolution model of RCT in China based on the data from railway container loading and unloading stations; however, RCT is a complicated transport activity, suggesting that there are still some limitations. This study only explores the present situation according to the data obtained from 2013 to 2020, and more continuous data will be obtained in future to evaluate the development pattern of RCT in China and explore the evolution mechanism behind it.

## Conclusion

This study focused on the RCT scale and center of gravity shift, RCT pattern evolution and agglomeration effect, and RCT functional differentiation as the main themes, while the temporal and spatial evolution law of RCT in China was described. The main conclusions are as follows:

1. The overall growth trend of scale is obvious, with flaky features, especially in the Bohai Rim region. The dual-center effect of the traffic scale in the BHEZ and CYEZ is obvious, and the YRDEZ has the estuary at the center and spreads in a ring shape. The transport direction changed, the northwest became the main export of railway container transport, and the transport center of gravity clearly moved to the north.
2. The attraction/occurrence of RCT is consistent in space distribution. Typical high-volume cities include Tianjin, Dalian, Chongqing, Kunming, and the railway container center station, with significant achievements having been made in the construction of the railway central station (27). The number of highly attractive cities and import cities increased from 8 to 50 and from 11 to 55, respectively. The agglomeration effect of railway container export and import is similar, essentially showing a trend of being higher in the northwest and lower in the southeast. The degree of agglomeration in the eastern, central, and western regions is vastly different, but it shows a balanced tendency. Both the degree of agglomeration and transport volume in the western region are higher. The RCT in BHEZ is the most balanced, and the CYEZ has a high concentration, but the transport proportion is low. The RCT in the PRDEZ needs to be strengthened, and the YRDEZ is dominated by railway container exports. Balancing regional freight differences will serve to promote the sustainable and healthy development of railway container freight.
3. China's freight transport in the north has obvious benefits, but its advantages are reduced, such as in Fuxin, Luliang, and Benxi. The attractive advantage of freight transport in the south is stronger and more obvious than that in the north. In the urban transport function, the various types of cities in the eastern and western regions can be ranked by quantity as comprehensive >export >import. RCT in most cities is relatively balanced. The scattered commodities in the western region are imported, and the number of import-type cities increased. The export-type in the eastern region increased, while the import-type decreased, which is linked to the industrial structure, with foreign trade as the main factor. All types of cities in the central region are stable, which is related to the economic structure of imports from the west, and exports to the east and west. Nearly half of China's cities have stable traffic functions, stable traffic supply, and demand relationships, and their industrial structure is continuously upgrading.

In order to boost China's freight transport to “road transport to railway transport revolution” and railway freight transport to “bulk cargo transport to container transport”, rationally adjust the functions of urban freight transport, and promote the healthy development of urban traffic, the authors put forward the following policy suggestions based on the research conclusions of this study: (1) fully understand the necessity of building a railway container transportation center station, and rationally plan the layout. Therefore, we should strengthen the inclination of the railway infrastructure and allocation of container resources in Tianjin, Dalian, Chongqing, and other high-traffic cities so as to strengthen the radiation driving effect of the central station on the region. Integrate freight resources of Yichun, Dandong, and other cities with small traffic volume in order to improve the efficiency of resource operation. Give full play to the dual-center effect of the Bohai Economic Zone and Chengdu–Chongqing Economic Zone and actively promote the construction of the transportation center. (2) According to the characteristics that the transportation center is concentrated in Henan and moved to the north, the investment and construction of Henan railway container transportation infrastructure should be appropriately strengthened, including optimizing railway lines and increasing handling stations. In view of the basic situation of China's railway container transportation “going from the north to the south”, it is necessary to strengthen the construction of the north–south railway network, the construction of key transportation channels, and the construction of high-quality railway logistics channels so as to achieve the balance of logistics transportation quality and efficiency. (3) In view of the change in the urban RCT function and according to the relationship between transportation supply and demand, maximize the advantages of regional transportation, promote the upgrading of the industrial structure in the central and western regions, and ensure the healthy development of China's RCT market. In addition, strengthening the connection with other modes of transportation and promoting the integration of railway containers into multimodal transport are crucial for future development of RCT.

RCHS data provide a new data source for evaluating the development of RCT in China. A more accurate analysis of the present situation of RCT is vital for exploring its driving mechanism and rationally allocating container resources in future. In future research, we will obtain more continuous data, explore the driving mechanism, and investigate the progress of China's railway container transport.

## Data availability statement

The data analyzed in this study is subject to the following licenses/restrictions. The data is obtained from the organization cooperating with the research group, and it is confidential. If necessary, you can contact the author to obtain the declassified data. Requests to access these datasets should be directed to ZB, bzz1118@dlmu.edu.cn.

## Author contributions

ZB and HL: data methodology. ZB: software and validation, formal analysis, writing—original draft preparation, and visualization. ZB and HK: writing—review and editing. HK and JY: supervision. HK: funding acquisition. All authors have read and agreed to the published version of the manuscript.
